# First insight into microbiome profile of fungivorous thrips *Hoplothrips carpathicus* (Insecta: Thysanoptera) at different developmental stages: molecular evidence of *Wolbachia* endosymbiosis

**DOI:** 10.1038/s41598-018-32747-x

**Published:** 2018-09-26

**Authors:** Agnieszka Kaczmarczyk, Halina Kucharczyk, Marek Kucharczyk, Przemysław Kapusta, Jerzy Sell, Sylwia Zielińska

**Affiliations:** 1 Department of Genetics and Biosystematics, Faculty of Biology, University of Gdansk, Wita Stwosza 59, 80-308, Gdansk, Poland; 2 Department of Zoology, Maria Curie-Sklodowska University, Akademicka 19, 20-033, Lublin, Poland; 3 Department of Nature Protection, Maria Curie-Sklodowska University, Akademicka 19, 20-033, Lublin, Poland; 4 Center for Medical Genomics – OMICRON, Jagiellonian University Medical College, Kopernika 7c, 31-034, Kraków, Poland; 5 Department of Bacterial Molecular Genetics, Faculty of Biology, University of Gdansk, Wita Stwosza 59, 80-308, Gdansk, Poland; 6 Phage Consultants, Partyzantow 10/18, 80-254, Gdansk, Poland

## Abstract

Insects’ exoskeleton, gut, hemocoel, and cells are colonized by various microorganisms that often play important roles in their host life. Moreover, insects are frequently infected by vertically transmitted symbionts that can manipulate their reproduction. The aims of this study were the characterization of bacterial communities of four developmental stages of the fungivorous species *Hoplothrips carpathicus* (Thysanoptera: Phlaeothripidae), verification of the presence of *Wolbachia*, *in silico* prediction of metabolic potentials of the microorganisms, and sequencing its mitochondrial COI barcode. Taxonomy-based analysis indicated that the bacterial community of *H*. *carpathicus* contained 21 bacterial phyla. The most abundant phyla were *Proteobacteria*, *Actinobacteria*, *Bacterioidetes* and *Firmicutes*, and the most abundant classes were *Alphaproteobacteria*, *Actinobacteria*, *Gammaproteobacteria* and *Betaproteobacteria*, with different proportions in the total share. For pupa and imago (adult) the most abundant genus was *Wolbachia*, which comprised 69.95% and 56.11% of total bacterial population respectively. Moreover, similarity analysis of bacterial communities showed that changes in microbiome composition are congruent with the successive stages of *H*. *carpathicus* development. PICRUSt analysis predicted that each bacterial community should be rich in genes involved in membrane transport, amino acid metabolism, carbohydrate metabolism, replication and repair processes.

## Introduction

Insects are by far the most diverse and abundant animal group, in numbers of species globally, in ecological habits, and in biomass^1^. They are chronically colonized by various microorganisms that are not overtly pathogenic and are often beneficial or even required by the insect host. These microorganisms colonize on the insect exoskeleton, in the gut and hemocoel, and within insect cells^2^. Bacterial communities are known to play important roles in many crucial aspects of their hosts life, e.g. nutrition, development, pathogen defense, community interactions and survival in harsh environments by metabolizing toxins^3–13^. Moreover, insects are also frequently infected by vertically transmitted symbionts that manipulate their reproduction^14^. The lack of vertical transmission through male hosts has led to the evolution of five commonly recognized manipulation schemes: feminization, parthenogenesis induction, early and late male killing, and cytoplasmic incompatibility^15^. One of the microorganisms that induce these alterations is *Wolbachia*. It is estimated to infect more than 65% of all insect species^16,17^. However, *Wolbachia* is just one of the known reproductive manipulators, others are *Cardinium*, *Rickettsia*, *Arsenophonus* and *Spiroplasma*^18^. *Cardinium* has been shown to induce cytoplasmic incompatibility, feminization and parthenogenesis, while *Rickettsia* can cause parthenogenesis and male killing. *Arsenophonus* and *Spiroplasma* can also induce male killing. All of induced phenomena lead to female biased reproduction, which can be beneficial for infected matrilines, but simultaneously facilitate drastic evolutionary trajectories of their hosts^15,19–21^. The wide range of phenomena in which bacteria are involved during insects life-cycle makes research focused on defining the microbiome profile of insects species (especially those exposed to harsh environmental conditions) of particular interest.

Analyses of bacterial communities associated with insects is facilitated by Next Generation Sequencing (NGS) of the 16S rRNA gene (e.g.^22–24^). Excluding bacteria, insects are known to represent more than half of the world’s biodiversity. A growing number of studies concern the analysis of bacterial communities associated with insects in a particular context, e.g. transfer of gut bacteria in social insects^25^, endosymbiosis and intracellular symbionts transmission^15,26^ as well as development of new strategies to prevent transmission of human pathogens^27^. Thrips on the whole have received relatively little attention regarding their bacterial communities. Previous studies have largely focused on the most frequently encountered bacterium (identified as a near-*Erwinia* species) within a single thrips species *Frankliniella occidentalis*^28–32^ with a few studies on other species (i.e. *Aptinothrips* species^33^, *Thrips tabaci*^34^, *Scirtothrips dorsalis*^35^, *Frankliniella fusca*^36^ and *F*. *tritici*^37^). However, nothing is known about the structure of bacterial communities associated with fungivorous thrips species.

*Hoplothrips carpathicus* occurs in central and northern Europe^38–41^. It belongs to the Phlaeothripidae family and the Phlaeothripinae subfamily of the insects order Thysanoptera. Fungivorous species of the Phlaeothripinae subfamily have a narrow maxillary canal and feed on mycelium or spores of fungi covering decaying wood, sucking the contents of their cells. *H*. *carpathicus*, like most of fungivorous species of this genus, lives in small cavities under the bark of dead trees or on/in fruiting bodies of different species of fungi. Their hidden lifestyle and haphazard spatial dispersion are the reasons of difficulties in observing them in their natural habitat. Females with fully developed wings (macropterous form) can be reliably collected by using IBL-2 screen traps hanging on 1.5 m high in deciduous forests^41^ (Supplementary Fig. S1) while the immature stages together with adults with or without wings can be collected by extracting from *Fomes fomentarius* (L.) Fr. (Basidiomycota) growing on different trees: beech (*Fagus sylvatica* L.), birch (*Betula pendula* Roth.), oak (*Quercus robur* L.) and Norway spruce (*Picea abies* (L.) H. Karst). The fruiting body of *F*. *fomentarius* provides many cavities, where *H*. *carpathicus* may live and find food. Recent studies have shown that biological compounds produced by *F*. *fomentarius* have many activities, such as antioxidant, anti-inflammatory, apoptotic, and anti-diabetic^42–46^. These compounds (e.g. triterpenoids) may be considered as toxins, especially for bacteria associated with fungivorous species. Bacterial communities associated with invertebrates which are the would-be colonizers need to overcome the presence of these substances through resistance or tolerance mechanisms.

In the present study we used NGS of the 16S rRNA gene to define whether the bacterial composition varies among the different developmental stages of *H*. *carpathicus*. We tested the hypothesis that a known endosymbiont might be present in the microbiome profiles. We also predicted the metabolic activity of the microorganisms associated with different developmental stages of *H*. *carpathicus*. Lastly, we sequenced the mitochondrial COI barcode of H. *carpathicus* to facilitate future molecular identification.

## Results

### COI sequence analysis

The barcode COI region of two specimens representing different developmental stages of *H*. *carpathicus* (pupa and adult) was sequenced. With an consensus length of 653 nucleotides, the sequences obtained contained no insertions, deletions or stop codons. Both sequences represented the same haplotype. Comparison with GenBank records and homology search shown high similarity of obtained *H*. *carpathicus* COI sequences with those obtained for other representatives of order Thysanoptera (≥86% similarity among the top 100 Blast hits on 100 subject sequences). All top 100 Blast hits were to Thysanoptera.

### General description of 16S rRNA gene sequencing results

For each developmental stage of *H*. *carpathicus*, we obtained >65 000 good quality 16S rRNA gene sequences, ranging between 66 241 for the first stage larvae (L1) and 295 697 for the imago stage (Im). More details for sequence data for each stage of *H*. *carpathicus* as well as the number of the observed OTUs and values of Chao1 index, are shown in Table 1. At least 352 OTUs, ranging from 352–1406, were observed in different developmental stages, which indicates that the microbial population is highly complex. Moreover, rarefaction analysis of the obtained data revealed trends indicating that sampling of microbial communities varied in their degree of completion by life stage (Supplementary Fig. S2). Diversity indices for each microbiome are reported in Supplementary Table S1.

**Table 1:** Summary of the sequencing data of microbial communities.

ID	No. of bacterial reads	Average length (bp)	No. of observed OTU’s	Chao1 Index
L1	66,241	446	352	412
L2	278,136	449	1164	1063
P	220,188	445	1281	1397
Im	295,697	447	1406	1277
Total	860,262	447	2541	1037

The ID abbreviations are defined in text. The numer of OTUs (operational taxonomic units) was generated at the 97% sequence similarity cut-off.

**Figure 1:**
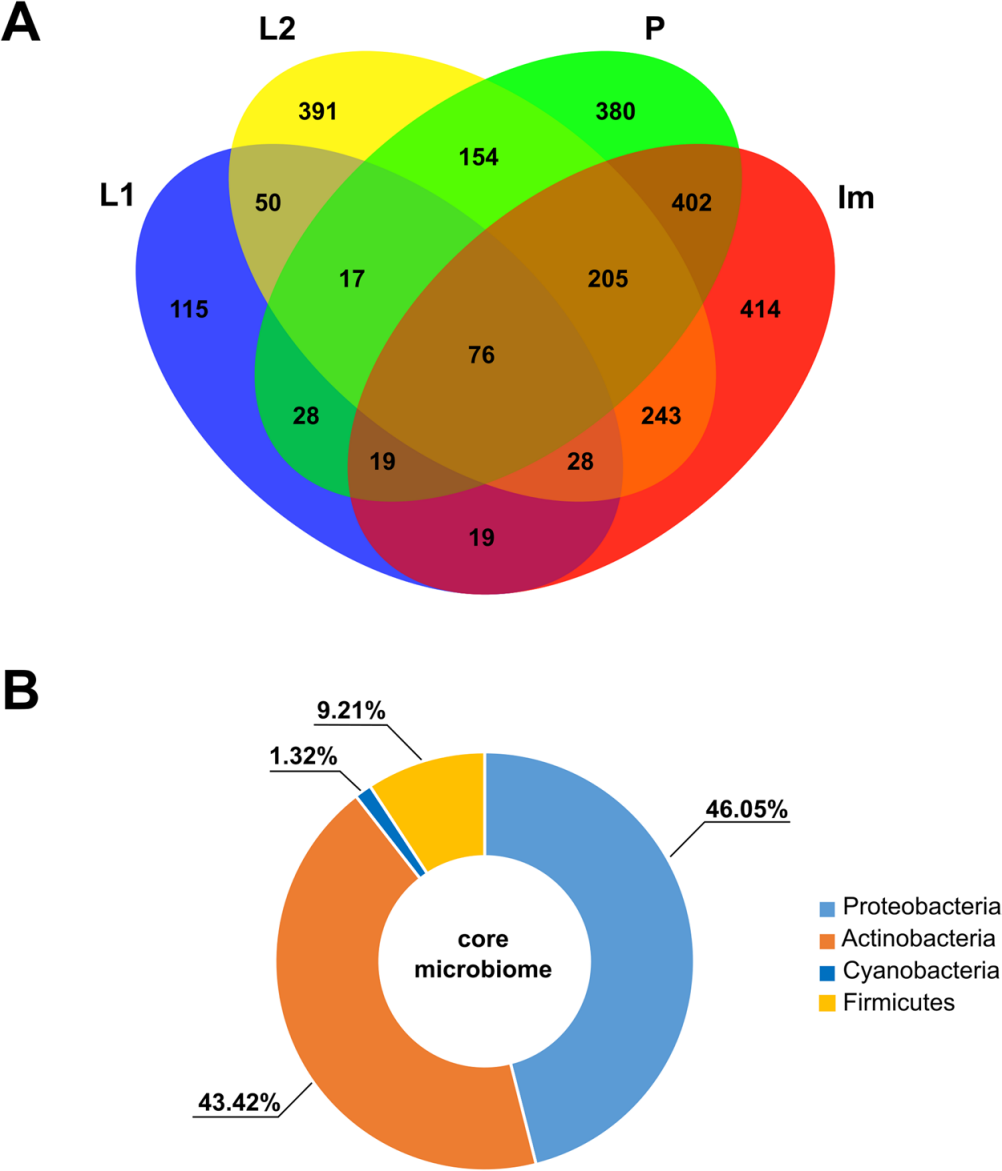
Analysis of OTUs at 97% similarity among different developmental stages of *H*. *carpathicus*. (**A**) Venn diagram showing overlaps of OTUs, in which 76 OTUs (core microbial community) were common for each tested stage. (**B**) Percentage distribution of core microbial community (76 OTU) at phylum level, identified in all developmental stages. Abbreviations: L1 – first-stage larva, L2 – second-stage larva, P – pupa, Im – adult.

The number of shared OTUs among developmental stages of *H*. *carpathicus* is shown as a Venn diagram (Fig. 1). We were able to classify 99.80% of sequences to phylum. Detailed taxonomic analyses on different ranks are available in supplementary data as sunburst charts (Supplementary Fig. S3) and also in a table (Supplementary Table S2).

### Microbial community composition

**Figure 2:**
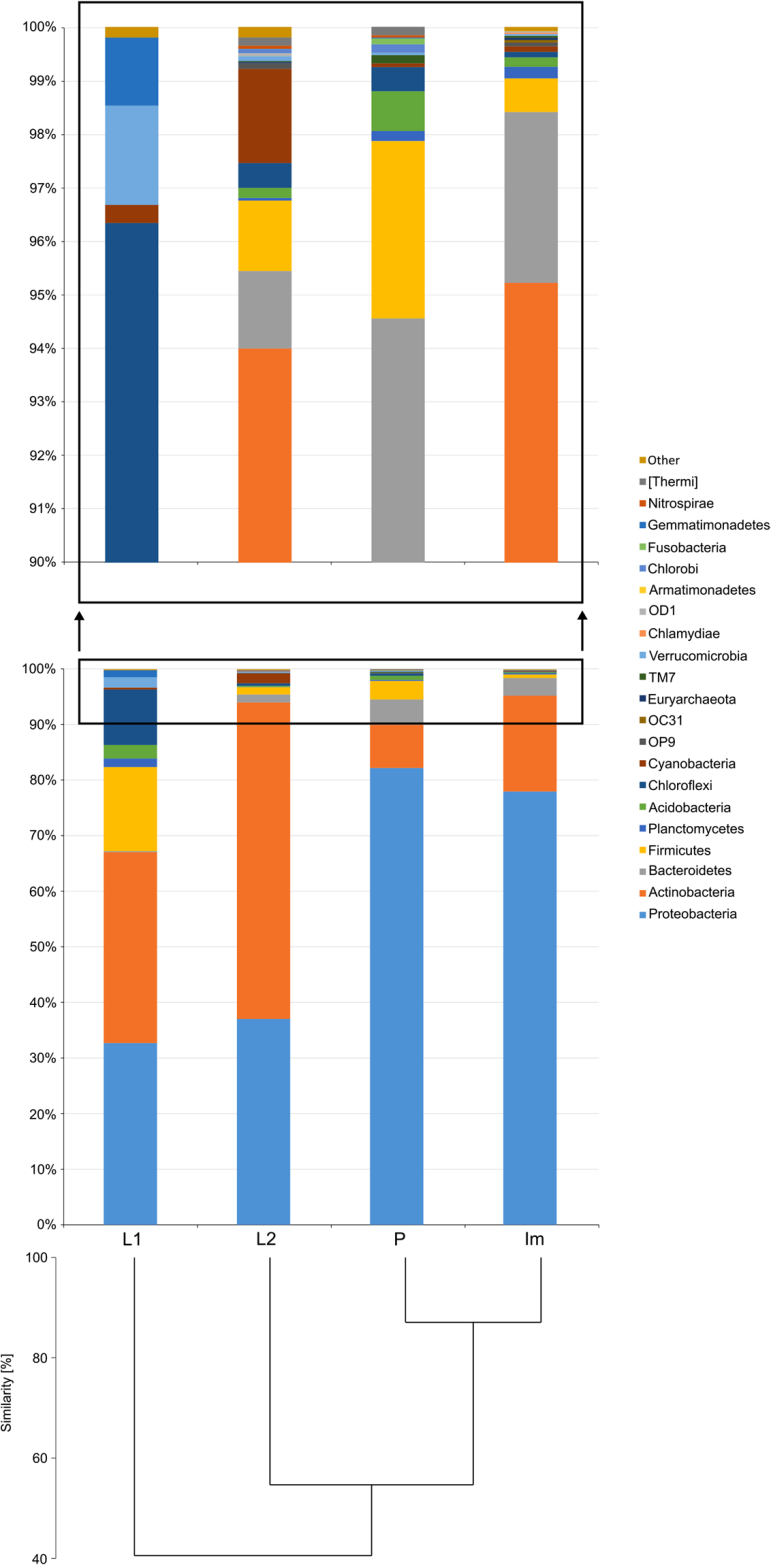
Abundance of bacterial 16S rRNA gene sequences at the phylum level with UPGMA clustering of *H*. *carpathicus* samples at different developmental stages according to community composition and structure. Abbreviations: L1 – first-stage larva, L2 – second-stage larva, P – pupa, Im – adult.

For all stages, analysis of microbial communities showed that >99.78% of the total reads were affiliated with *Bacteria* (Supplementary Fig. S3 and Supplementary Table S2). The remaining percentage comprised *Archaea* and unassigned records. The four microbiomes contained 21 phyla (Fig. 2, Supplementary Fig. S3 and Supplementary Table S2).

The most abundant phyla across all stages tested were *Proteobacteria*, *Actinobacteria*, *Bacterioidetes* and *Firmicutes* with each comprising a different share of the microbiome depending on developmental stage. In each developmental stage, these four phyla comprised >80.00% of the total microbial sequences obtained. Separately, *Proteobacteria* comprised on average 57.49% (32.71% in L1 to 82.20% in the pupal stage – P), *Actinobacteria* on average 29.07% (7.77% in P to 56.94% in the second stage larvae – L2), *Firmicutes* on average 5.10% (0.63% in Im to 15.13% in L1), and *Bacteroidetes* on average 2.36% (0.20% in L1 to 4.58% in P) of the total reads (Fig. 2 and Supplementary Table S2). SIMPER analysis showed that the share of *Proteobacteria*, *Actinobacteria* and *Firmicutes* was primarily responsible for the difference between samples (Table 2). The bacterial structure of all-but-one developmental stage pair differed significantly (Table 2). Only in the pupa-imago pair did the microbial communities not differ significantly (χ^2^ = 7.20, df = 21, *P* > 0.05).

**Table 2:** Average dissimilarity in microbial community structure and values of Chi-square test.

	Average dissimilarity (%)					
	L1-L2	L1-P	L1-Im	L2-P	L2-Im	P-Im
*Proteobacteria*	2.17	24.74	22.64	22.57	20.47	2.11
*Actinobacteria*	11.30	13.28	8.56	24.58	19.85	4.73
*Firmicutes*	6.90	5.91	7.25	0.99	0.35	1.34
*Chloroflexi*	4.76	4.76	4.94	0.00	0.18	0.18
*Bacteroidetes*	0.62	2.19	1.50	1.57	0.87	0.70
*Verrucomicrobia*	0.88	0.91	0.92	0.02	0.03	0.01
*Acidobacteria*	1.13	0.86	1.14	0.28	0.01	0.28
*Planctomycetes*	0.74	0.67	0.66	0.07	0.08	0.02
*Gemmatimonadetes*	0.63	0.63	0.64	0.00	0.01	0.01
*Cyanobacteria*	0.71	0.14	0.12	0.84	0.83	0.02
[Thermi]	0.07	0.08	0.00	0.00	0.07	0.08
*Chlorobi*	0.04	0.08	0.00	0.04	0.04	0.08
TM7	0.01	0.08	0.02	0.07	0.01	0.06
*Fusobacteria*	0.00	0.06	0.00	0.05	0.00	0.06
*Nitrospirae*	0.03	0.02	0.00	0.01	0.03	0.02
*Armatimonadetes*	0.00	0.00	0.00	0.00	0.00	0.00
*Chlamydiae*	0.00	0.00	0.02	0.00	0.02	0.02
*Euryarchaeota*	0.01	0.00	0.02	0.01	0.01	0.02
OC31	0.00	0.00	0.03	0.00	0.03	0.03
OD1	0.03	0.00	0.01	0.03	0.02	0.01
OP9	0.05	0.00	0.04	0.05	0.02	0.04
Other	0.00	0.10	0.06	0.10	0.06	0.04
Overall	30.10	54.49	48.52	51.31	42.98	9.82
	**Chi-square test (df** = **21)**					
χ^2^ value	34.75	64.28	56.26	59.58	39.02	7.20
*p* value	*p* < 0.05	*p* < 0.001	*p* < 0.001	*p* < 0.001	*p* < 0.01	*p* = 1.00

The most abundant classes among bacterial communities of all development stages were *Alphaproteobacteria*, *Actinobacteria*, *Gammaproteobacteria*, *Betaproteobacteria* and *Bacilli* accounting for >72.00% of the total reads (Supplementary Fig. S3 and Supplementary Table S2). For pupa and adult stages the most dominant class was *Alphaproteobacteria*, and the most dominant order among *Alphaproteobacteria* was *Rickettsiales* (69.95% and 56.11%, respectively). The most dominant family was *Rickettsiaceae* and among that family the most dominant genus was *Wolbachia*, which comprised 69.95% and 56.11% of sequences in pupa and adult respectively. In the L2 larva microbiome, the most dominant genus was *Tsukamurella* (phylum *Actinobacteria*), which accounted 45.26% of total microbial reads. Among first-stage larva *Actinobacteria* was the most dominant phylum, which accounted 34.34%, and order *Actinomycetales* consisted 28.56% of that phylum.

Similarity analysis of bacterial communities based on UPGMA clustering of samples at different developmental stages (Fig. 2) showed that changes in microbiome composition are congruent with the successive stages of *H*. *carpathicus* development. Microbiome profiles of pupa and adult are the most similar, whereas the bacterial community composition of first-stage larva was less similar to those associated with other stages tested.

**Figure 3:**
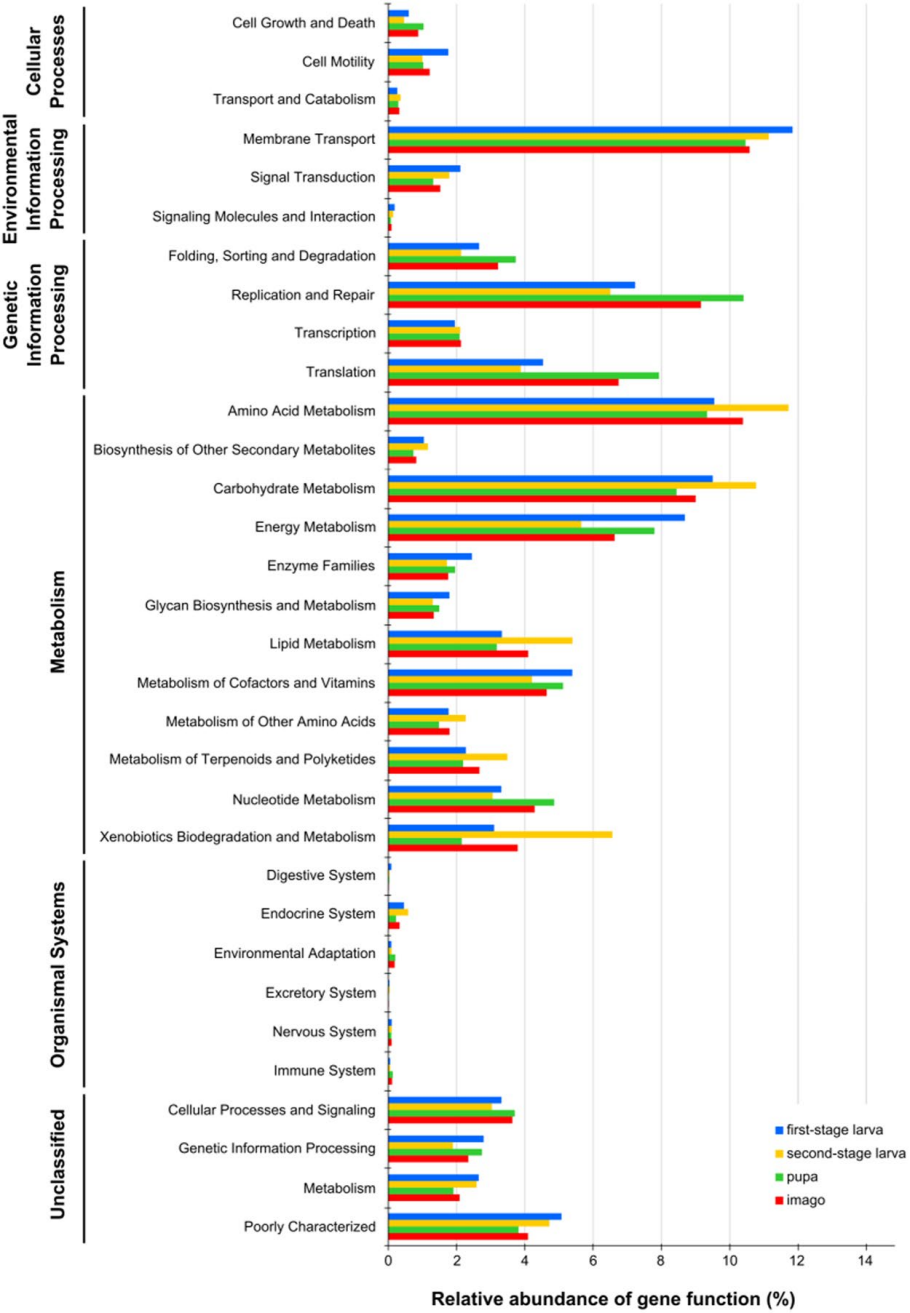
Inferred functions of bacterial communities associated with different developmental stages of *H*. *carpathicus*. All of the predicted KEGG metabolic pathways are shown at the second hierarchical level and grouped by major functional categories.

PICRUSt analysis predicted functional potentials of the bacterial community associated with different developmental stages. In all of the tested stages bacterial communities seem to be rich in genes involved in membrane transport, amino acid metabolism, carbohydrate metabolism, and replication and repair processes (Fig. 3). Predicted genes involved in membrane transport are thought to be connected with ATP-binding cassette transporters (ABC transporters), the phosphoenolpyruvate (PEP)-dependent phosphotransferase system (PTS system) and bacterial secretion system (not shown).

In both larval stages, predicted genes thought to be involved in metabolism of terpenoids and polyketides, and xenobiotics biodegradation and metabolism were relatively abundant. Based on OTU abundance, predicted genes thought to be involved in xenobiotics biodegradation and metabolism would be increased in L2 larvae relative to L1. In the pupa-associated bacterial community several functional categories (i.e. carbohydrate and lipid metabolism, signal transduction, and membrane transport) are decreased compared to larval stages. However, relative abundance of predicted genes thought to be involved in energy and nucleotide metabolism, replication and repair, and translation is increased in the pupa. In the adult stage, several predicted pathways should be more abundant than in pupal stage based on differential OTU relative abundance (e.g. amino acids, carbohydrate and lipid metabolism, as well as xenobiotics biodegradation and metabolism) (Fig. 3).

## Discussion

Identification of most thrips to a species level is difficult due to their small size, subtle morphological differences^47,48^, high intraspecific polymorphism^49^, sexual dimorphism^50^, and the presence of cryptic species and species complexes^48,51,52^. Molecular identification of Thysanoptera species is desirable as it overcomes these complexities. Therefore, in this study the barcode COI region was sequenced for the first time for *H*. *carpathicus*. This species can now be included in future phylogenetic analyses focused on resolving relationships among thrips species. Nevertheless, the main part of the present study was the characterization of developmental-stage-specific microbiomes associated with this fungivorous thrips species.

Microorganisms display a wide diversity of specialized interactions with their insect hosts. These associations are ubiquitous and often beneficial to the insect^53^. Nonetheless, most studies on insect microbiomes have focused on the gut, most frequently for termites^54–56^, ants^57–59^, fire bugs^60–62^, fruit flies^63–65^, beetles^66–68^, and bees^69–72^. Thrips, thus far, have received little attention regarding these important microbiome-host interactions. Previous work has focused largely on the bacterium identified as near-*Erwinia* species and frequently encountered in *Frankliniella occidentalis*, *F*. *fusca* and *Thrips tabaci* (e.g.^34,36,73^). Analyses of thrips microbiomes with NGS techniques is much more limited (but see^35,37^).

This study resolves the complex microbial population structure of different developmental stages of *H*. *carpathicus*. In several studies of insect’s microbiome, authors recommend surface sterilization prior to DNA extraction^74,75^. The individuals tested here were rinsed three times in sterile distilled water without soaking in ethanol. However, Hammer *et al*.^76^ found that surface sterilization did not change bacterial community structure as compared to unsterilized specimens, which may be due to most of the bacteria residing inside the insect body relative to its surface. In the present study, we investigated the structure and relationships of bacterial communities associated with four developmental stages of *H*. *carpathicus*, without division into endo- and ectomicrobiome.

The present study also allowed us to track changes in the microbiome profiles associated with the species development. Using the UPGMA clustering method, we found that microbial communities of pupa and adult are the most similar (~90.00%) whereas microbiome associated with first-stage larva is less similar (~40.00%). This observation is consistent with numbers of observed OTUs – 352 in L1 larvae and ~3X higher in subsequent developmental stages. This is indicative of an increasingly complex bacterial community as development progresses.

In all tested developmental stages of *H*. *carpathicus* the most dominant phyla were *Proteobacteria*, *Actinobacteria*, *Bacteroidetes*. and *Firmicutes*. This is not surprising and has been found in other insects^77–79^. Although studies focused on bacterial communities associated with Thysanoptera species are limited, recent studies confirmed that *Proteobacteria* and *Actinobacteria* are the most abundant phyla in bacterial community of other thrips species, i.e. *Scirtothrips dorsalis*^35^ and *Thrips palmi*^79^. *Proteobacteria* comprises ~60% of bacteria detected in *S*. *dorsalis* and similar abundance was observed *in H*. *carpathicus* (~57%). In *T*. *palmi* this phylum was slightly less abundant and comprises ~50% of detected bacteria. The average abundance of *Actinobacteria* was similar for *S*. *dorsalis* and *H*. *carpathicus* (~33% vs. ~29%) while it was lower in *T*. *palmi* (~20%). *Firmicutes* and *Bacterioidetes*, although less abundant than *Proteobacteria* and *Actinobacteria*, in all tested in here stages of *H*. *carpathicus*, are also listed among dominant phyla in *T*. *palmi*^79^. *Firmicutes* comprises ~15% of bacteria detected in this species. Similar abundance of this phylum was identified in L1 sample, while in the case of other samples tested this phylum was less abundant and comprised no more than 4%. Differences in abundance was also observed in the case of *Bacteroidetes*. This phylum comprised ~10% of bacteria detected in *T*. *palmi*, but comprised no more than 5% of bacteria in *H*. *carpathicus*. The proportion of most abundant phyla is not stable during ontogenesis. In first- and second stage larvae *Actinobacteria* and *Proteobacteria* were the most dominant, but in pupa and adult the percentage of these two phyla changed significantly. The number of *Actinobacteria* decreased >2-fold, while the amount of *Proteobacteria* increased >2-fold. Higher relative abundance of *Proteobacteria* in adults than in larvae has been noted other insects^74,80^. Moreover, in pupa and adult of *H*. *carpathicus*, the abundance of *Bacteroidetes* declined. Studies comparing bacterial communities associated with insect’s successive life stages remains limited (~10 published papers in the last 6 years)^66,74,79,81–87^. Therefore, extrapolating these results to other studies is difficult. However, it is possible that change in ratio of main bacterial phyla is connected with pupation, when the gut is remodeled. During the transition from the second instar to adult in the propupa and pupa, many imaginifugal characters are broken down and reformed. According to Parker *et al*.^88^, the length of the midgut decreases greatly during development and the gut epithelium becomes pycnotic and is replaced by new gut cells. In adults midgut forms a long tube divided into three different histological regions^89^. The larval pygidial gland, which is typical imaginifugal structure, degenerates in the pupal stage^88^. These changes may indirectly impact the microbial communities and thus cause changes in bacterial composition.

We identified five genera: *Agrobacterium*, *Erwinia*, *Methylobacterium*, *Pseudomonas*, *Serratia*, which have previously been associated with other thrips species. *Pseudomonas*, *Serratia* and *Erwinia* have been identified internally from *F*. *occidentalis* and *T*. *tabaci*^30,32,34,35,73^, while *Agrobacterium*, *Methylobacterium* and *Pseudomonas* have been identified from *S*. *staphylinus*^90^ and *S*. *dorsalis*^35^. Analogous to Dickey *et al*.^35^, in our study DNA was extracted from whole body of tested individuals and therefore, it is impossible to clearly distinguish internal symbionts and those associated with the surface of the insect. Based on the previous reports of Yamoah *et al*.^90^, we think that *Agrobacterium*, *Methylobacterium* and *Pseudomonas* genera may be associated with exoskeleton of *H*. *carpathicus*.

It is known that *F*. *fomentarius* has antimicrobial, antioxidant, anti-inflammatory, apoptotic, and anti-diabetic activities^42–46,91^. Specifically, Kolundžíć *et al*.^92^ tested antimicrobial activity of *F*. *fomentarius* extracts of different polarity using nine different laboratory strains of gram negative and positive bacteria, respectively (*Staphylococcus aureus*, *Staphylococcus epidemidis*, *Micrococcus luteus*, *Bacillus subtilis*, *Enterococcus feacalis*, *Escherichia coli*, *Klebsiella pneumoniae*, *Pseudomonas aeruginosa* and *Salmonella abony*). They reported higher antimicrobial activity from methanol and aqueous extracts than cyclohexane and dichloromethane extracts, but this activity was lower compared to semi-synthetic antibiotics (ampicillin and amikacin, respectively). In total, they reported significant antimicrobial activity of *F*. *fomentarius* extracts against nine bacterial strains. Their results and other studies suggest that the antimicrobial activity results from elevated polyphenols and β-glucan. Hence, based on these results and other studies^93–96^ polyphenols and β-glucan could be connected with significant antimicrobial activity. As we mentioned in Introduction, such compounds may be considered as toxins and bacteria associated with invertebrates which are the would-be colonizers of *F*. *fomentarius* fruiting bodies need to overcome the presence of these substances through resistance or tolerance mechanisms. While we did not distinguish internal and external bacteria on *H*. *carpathicus*, any in latter category should exhibit properties that make possible to survive under such conditions.

The present study concerns the activities of the most dominant microbial groups, as well as identification of predicted metabolic functions of the bacterial community associated with *H*. *carpathicus*. PICRUSt analysis showed, that in microbial communities associated with all of the tested stages genes involved in membrane transport, amino acid metabolism, carbohydrate metabolism, and replication and repair processes should be elevated (Fig. 3). Higher relative abundance of predicted genes involved in membrane transport (especially those connected with ABC transporters) might be related to antibiotic resistance, because ATP-binding cassette (ABC)-type multidrug transporters use a free energy of ATP hydrolysis to pump drugs out of the cell^97^. In second-stage larvae, which intensively feed on polypore tissue, the microbiome was more enriched for predicted genes thought to be involved in metabolism of terpenoids and polyketides, and xenobiotics biodegradation and metabolism. Analysis of bacterial community associated with pupae showed that several functional categories are predicted to be reduced, such as those thought to be involved in carbohydrate and lipid metabolism, signal transduction, and membrane transport. Genes thought to be involved in energy and nucleotide metabolism, replication and repair, as well as translation pathways should be elevated in the pupae – the stages which do not feed and move, suggesting a mechanism by which bacteria extract energy for surviving inside pupal case. At the adult stage, a higher abundance of several pathways was predicted, such as amino acid, carbohydrate and lipid metabolism. Nevertheless, the *in silico* predicted functions need to be validated *in vitro* in future studies.

Genes thought to be involved in metabolism of terpenoids and polyketides, as well as xenobiotics and biodegradation metabolism are predicted by our results to be elevated in adults. This suggests a mechanism by which bacteria associated with adult *H*. *carpathicus* could tolerate triterpenoids, the major class of secondary metabolites produced by *F*. *fomentarius*’ fruting body (~75.00%)^98^. Our OTU-to-gene-to-pathway prediction scheme identified also polysaccharide metabolism as important. It would be interesting to test whether specific members of microbiome are indeed metabolizing some of α- and β-glucans produced by *F*. *fomentarius*^98,99^. Among α-glucans presence in fungi are cellulose and chitin^98^. *H*. *carpathicus* feeding on *F*. *fomentarius* needs a way to process these α-glucans and to access to intercellular fungal nutrients. The present study predicts an association between *Tsukamurella* and cellulose degradation. This genus was identified as dominant in intensively feeding second-stage larvae and suggests a mechanism by which L2 larvae might process cellulose. Microbiome studies of other wood and fungus associated insects^100,101^ provide anecdotal confirmation of this suggestion. Other bacterial genera: *Staphylococcus*, *Bacillus*, *Sphingomonas*, *Brevibacterium* and *Chryseobacterium*, which we identified in this study, have also been linked to cellulose degradation^102–104^. Interestingly, the genus *Methylobacterium* was found in all developmental stages of *H*. *carpathicus*. Some species of *Methylobacterium* are facultative methylotrophic bacteria, and have been reported mostly from soil^90^, but also in plants^105^. They can use a variety of organic substrates with carbon-carbon bonds as sources of carbon and energy^106^. The role of *Methylobacterium* in *H*. *carpathicus* might be similar to that suggested by studies of leafhopper and weevil, which also feed on a complex carbon source^90,105^. Other predicted functions of the *H*. *carpathicus* bacterial community were determined according to comprehensive analysis of the functional microbiome of arthropods^107^ where *Agrobacterium*, *Methylobacterium* and *Serratia* were joined together within single group of microorganisms containing nitrogen fixing and anaerobic metabolic traits.

Beside general insight into the bacterial community associated with *H*. *carpathicus*, this study identified the presence of known endosymbionts. In previous studies of Thysanoptera species it has been argued that the bacteria are facultative and acquired from the environment through feeding^28,34,73^. Nevertheless, detailed analyses focused on bacterial endosymbionts (especially *Cardinium* and *Wolbachia*) showed that vertically transmitted symbionts are present among thrips species^108–111^. Our identification of *Wolbachia*, often described as a ‘master manipulator’^112^, is particularly interesting. This bacterium can eliminate males, turn them into females, sterilize uninfected females or behave as a mutualistic symbiont^113^. *Wolbachia* has been detected both in arrhenotokous and thelytokous thrips species^108,109,111^ although the frequency of this reproductive manipulators in Thysanoptera is unknown. The absence of *Wolbachia*, identified by conventional PCR, has been shown for three thrips: *Frankliniella occidentalis*^108^, *Thrips tabaci*^108,114^ and *Heliothrips haemorrhoidalis*^115^. Nevertheless, recent studies have demonstrated that conventional PCR can fail to detect low-level infections^116^. In the present study, *Wolbachia* was shown to be the most dominant genus in pupa and adult stages of *H*. *carpathicus* (69.95% and 56.11%, respectively), while in first and second larval instars it occurred at a lower frequency (1.25% in L1 and 10.67% in L2, respectively). The reason of observed disproportion is unknown. It could be that *Wolbachia* infection increased with development or is biased based on sex. In our study both males and females were seen but only adult females were used in microbiome characterization and the sex of non-adults characterized is impossible to determine. We also could not calculate the sex ratio because the specimens of all developmental stages were selected successively and the breeding was finished when only adults, mainly macropterous females, left the fruiting body of *F*. *fomentarius*. The presence of both sexes indicates arrhenotokous reproduction, where females develop from fertilized eggs and males from unfertilized ones. In this study, the presence of *Wolbachia* in a fungivorous thrips species *H*. *carpathicus* has been identified for the first time. Nevertheless, its impact on the host reproduction system remains unclear and follow-up analyses are planned.

In conclusion, this paper presents data of bacterial community analysis of *H*. *carpathicus* with the use of NGS 16S rRNA sequence data, which allowed us to nearly fully characterize its microbiome. Moreover, it is the first report of *Wolbachia* endosymbiosis in this thrips species. Additionally, the sequenced COI fragment of *H*. *carpathicus* may be useful to identify all developmental stages of this species using barcoding approach and can be applied in further phylogenetic studies. Finally, the results obtained raise new and important questions regarding the role of *Wolbachia* and *Tsukamurella* in *H*. *carpathicus* biology.

## Methods

### Sample collection

Fruiting bodies of *F*. *fomentarius* were collected in Roztocze National Park, Białowieża NP and Polesie NP in summer and autumn 2016. A single fruiting body of *F*. *fomentarius*, which was colonized by *H*. *carpathicus*, was collected on 19^th^ of November 2016 in Białowieża Primeval Forest (north-eastern Poland, geographical coordinates: 52°43′36.7″N and 23°47′22.7″E). Branches of dead oak were the host for this fungus. Singular specimens of *H*. *carpathicus* were extracted from the fruiting body using Tullgren funnel. It was subsequently placed into a plastic box with a damp paper towel at the bottom and stored in 24/10 °C, 16/8 h (L/D) conditions. The breeding was conducted in Department of Zoology Maria Curie-Sklodowska University in Lublin until January 23, 2017 when all adults left the fungus. During the breeding period, four developmental stages of *H*. *carpathicus* were successively selected: intensively feeding first and second larval instars (L1 and L2, respectively) and adults (Im), and pupae (P) that do not feed. Among adults, females had fully functional wings (macropterous form) and were more numerous, while males were wingless (apterous form) and less numerous. Therefore, for further analyses only adult females were used. Every stage was separately placed into tubes and stored in −30 °C. Afterwards the tubes with insects were sent for further analyses to the Department of Genetics and Biosystematics, University of Gdansk. Some specimens of all developmental stages (first and second larval instars, propupae, pupae, females with fully functional wings and wingless males) were mounted in Canada Balm. The examined materials are deposited in the collection of Department of Zoology, Maria Curie-Sklodowska University in Lublin.

### DNA extraction

Due to the small sizes of individuals (<2 mm), DNA was extracted from the whole insects at different developmental stages by the Sherlock AX Purification Kit (A&A Biotechnology). Insects were rinsed three times in sterile distilled water prior to DNA extraction without soaking in ethanol. To avoid cross contamination of the samples, the process was performed with sterile equipment. The quantity and quality of the extracted DNA were evaluated by using a Nano Drop ND-1000 spectrophotometer (NanoDrop Technologies). After extraction, the DNA was stored at −20 °C until further use. Twelve samples consisting of genetic material isolated from first and second larval instars, pupae and adults (one individual per DNA isolate and three isolates per developmental stage) were used for microbiome analyses.

### COI mitochondrial marker amplification and sequencing

The 5′ COI gene fragment was amplified for two individuals of *H*. *carpathicus* (pupa and adult), using universal primer set: HCO-2198 – GGTCAACAAATCATAAAGATATTGG and LCO-1490 – TAAACTTCAGGGTGACCAAAAAATCA^117^. PCR reactions were performed in 20 µL volume containing 0.8x JumpStart *Taq* ReadyMix (1 U JumpStart Taq DNA polymerase, 4 mM Tris-HCl, 20 mM KCl, 0.6 mM MgCl_2_, 0.08 mM dNTP; Sigma-Aldrich, Germany), 0.4 µM of forward and reverse primers and ~100 ng of DNA. The COI gene fragment was amplified under conditions as follows: initial denaturation at 94 °C for 5 min followed by 35 cycles of 94 °C for 1 min, 51.2 °C for 1 min and 72 °C for 1 min and ending with 72 °C for 5 min. All PCR products were purified by alkaline phosphatase and exonuclease I (Thermo Scientific, USA) treatment according to manufacturer’s protocols and sequenced with a BigDye^TM^ terminator cycle sequencing kit (Applied Biosystems, USA) at Macrogen (Amsterdam, Netherlands).

### 16S rRNA gene amplification and sequencing

The V3-V4 hypervariable regions of bacterial 16S rRNA gene region were amplified using the following primer set: 341F-CCTACGGGNGGCWGCAG and 785R-GACTACHVGGGTATCTAATCC. The targeted gene region has been shown to be suitable for Illumina sequencing^118^. Each library was prepared with a two-step PCR protocol based on Illumina’s “16S metagenomic library prep guide” (15044223 Rev. B), NEBNext® Q5 Hotstart High-Fidelity DNA polymerase (New England BioLabs Inc.) according to the manufacturer’s protocol using Q5® Hot Start High-Fidelity 2X Master Mix (NEBNext - New England BioLabs, PCR under the following conditions: 98 °C for 30 sec for initial denaturation of the DNA, followed by 25 cycles of 98 °C for 10 sec, 55 °C for 30 sec, 72 °C for 20 sec and additionally 72 °C for 2 min), and the Nextera Index kit (2 × 250 bp). Paired-end (PE, 2 × 250 nt) sequencing with a 5% PhiX spike-in was performed with an Illumina MiSeq (MiSeq Reagent kit v2) at Genomed, Warsaw, Poland; following the manufacturer’s run protocols (Illumina, Inc., San Diego, CA, USA). The automatic primary analysis and the de-multiplexing of the raw reads were performed on the MiSeq machine, with the use of MiSeq Reporter (MSR) v2.6 (16S Metagenomics Protocol).

### Sequencing data analysis and statistical analysis

The COI sequences were edited and corrected (primer removal and alignment trimming) using BioEdit software^119^. Comparison with GenBank records and homology search was carried out on 9^th^ of November 2017 using megablast algorithm^120,121^ and the Nucleotide database.

The data obtained for the 3 independent DNA extractions for each developmental stage of *H*. *carpathicus* were merged and considered as one sample in taxonomic analyses. The intension of this approach was to obtain a more reliable view into the “average” bacterial communities’ structure. The exact age of insects was not determined, because particular developmental stages were successively collected from fruiting body to avoid disturbing the colony excessively during breeding. Adults age have been estimated to be ~1 month.

The samples were processed and analyzed using the Quantitative Insights Into Microbial Ecology (QIIME, version 1.9.1) pipeline^122^. Paired-end reads from MiSeq sequencing were quality trimmed and joined with PANDAseq version 2.8^123^ with a quality threshold of 0.9. The sequences that did not meet the quality criteria were removed from further analysis (mean quality >20). Chimeric reads detection was performed with VSEARCH, version 1.7.0^124^, an open-source replacement of USEARCH software. Clustering of operational taxonomic units (OTUs) at 97% similarity was performed by using the uclust method, version 1.2.22q^125^. OTUs were assigned to taxa using the GreenGenes release 13.5 database as the reference^126^, with the taxonomy assignment tool PyNAST^127^. The Biological Observation Matrix (BIOM) table was used as the core data for downstream analyses^128^. Any sequences that were classified as Mitochondria or Chloroplast, as well as singletons, were filtered out of the dataset. Trends in rarefaction analysis of the obtained data (plateau curves) have been used for estimation of completeness of microbial communities sampling. Based on clusters, the diversity indices were estimated, including the Chao1, PD (a quantitative measure of phylogenetic diversity), Shannon, and Simpson indices and also the number of observed OTUs. The Chao1 index is a non-parametric richness estimator that calculates the minimal number of OTUs present in a sample. Shannon index measures the average degree of uncertainty in predicting the identity of an individual sequence chosen at random. Its value increases as the number of species increases and as the distribution of individuals among the species becomes more even. The Simpson index is the probability of sampling successively at random two individuals of the same species. It varies from 0 to 1 and increases as the number of species increases and as the distribution of individuals among the species becomes less even^129^. Comparison of the bacterial community structure was performed with the use of UniFrac^130^ and Emperor^131^. A membership Venn diagram was computed using the MetaCoMET^132^ web platform to determine the specific and shared OTUs across the developmental stages of *H*. *carpathicus*. Similarity percentage (SIMPER) analysis^133^ was performed to calculate the average dissimilarities in bacterial community structures between different developmental stages of *H*. *carpathicus* and to assess which phylum was responsible for the observed differences. The differences in bacterial community structures were tested by the χ^2^ test. Statistical analyses were performed using PAST 3.16^134^ and PopTools 3.2^135^ software.

All obtained sequential data were deposited in open-source databases. Both COI sequences have been deposited in GenBank under accession numbers MG491887 and MG491888 (pupa and adult, respectively). NGS raw reads were deposited under study accession number PRJEB22873 in ENA – the European Nucleotide Archive.

The software PICRUSt (Phylogenetic Investigation of Communities by Reconstruction of Unobserved States^136^) was used to infer metabolic capacity of the microbiome from the 16S sequences. PICRUSt functional inference is implemented in two steps. First, a reference phylogenetic tree is constructed from the Greengenes database^126^ and gene contents are assigned to nodes in the tree if sequenced genomes are available, or otherwise predicted using ancestral state reconstruction algorithms^136^. Representative sequences from OTUs derived from experimental data and associated with Greengenes identifiers are normalized by 16S rRNA gene copy number and then mapped to the corresponding Greengenes identifiers in the reference tree. The final result is an annotated table of predicted gene counts per sample. Predicted annotations can be linked with PICRUSt software to the Kyoto encyclopedia of genes and genomes (KEGG) orthology (KO) accession numbers^137^.

## Electronic supplementary material

Supplementary Fig. S1

Supplementary Fig. S2

Supplementary Table S1

Supplementary Table S2

Supplementary Fig. S3

## Data Availability

The NGS data sets were deposited at the European Nucleotide Archive (ENA) under the Accession number PRJEB22873. COI barcode sequences were deposited in GenBank database under the Accession numbers MG491887 and MG491888. Sunburst charts of the relative abundance of microbial 16S rDNA sequences at different taxonomic levels are available for download from the Dropbox repository (https://www.dropbox.com/sh/2pseqdq09cgi9kg/AAAH_tcwOAF0aL1xhGStgJ_ja?dl=0).
