# Supplementary material for: First insight into microbiome profile of fungivorous thrips Hoplothrips carpathicus (Insecta: Thysanoptera) at different developmental stages: molecular evidence of Wolbachia endosymbiosis
Source: Sci Rep. 2018 Sep 26;8:14376. doi: 10.1038/s41598-018-32747-x (PMC6158184; doi:10.1038/s41598-018-32747-x)
Supplement: Supplementary file 1 — Supplementary Fig. S1 [file 41598_2018_32747_MOESM1_ESM.pdf]

First insight into microbiome profile of fungivorous thrips *Hoplothrips carpathicus* (Insecta: Thysanoptera) at different developmental stages: molecular evidence of *Wolbachia* endosymbiosis

Agnieszka Kaczmarczyk, Halina Kucharczyk, Marek Kucharczyk, Przemysław Kapusta, Jerzy Sell, Sylwia Zielińska

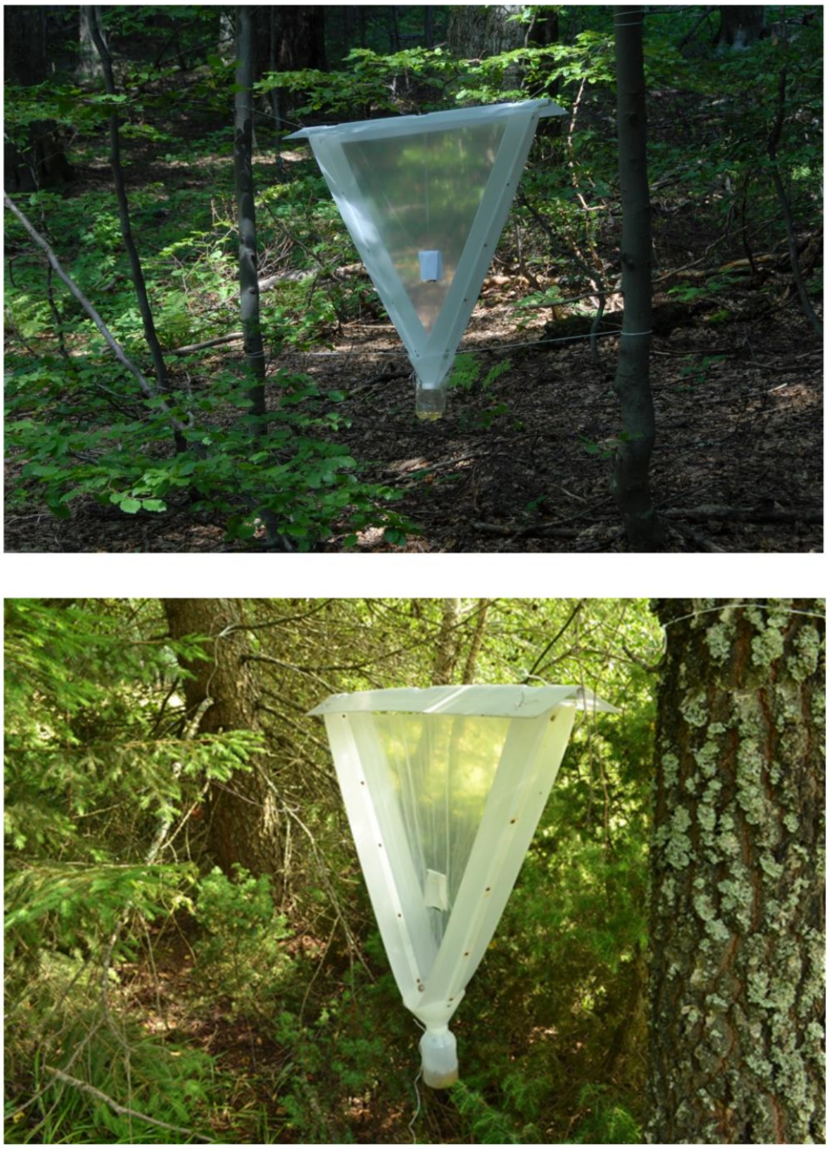

Supplementary Fig. S1. An IBL-2 type screen trap used to catch insects.
