# Supplementary material for: First insight into microbiome profile of fungivorous thrips Hoplothrips carpathicus (Insecta: Thysanoptera) at different developmental stages: molecular evidence of Wolbachia endosymbiosis
Source: Sci Rep. 2018 Sep 26;8:14376. doi: 10.1038/s41598-018-32747-x (PMC6158184; doi:10.1038/s41598-018-32747-x)
Supplement: Supplementary file 2 — Supplementary Fig. S2 [file 41598_2018_32747_MOESM2_ESM.pdf]

First insight into microbiome profile of fungivorous thrips *Hoplothrips carpathicus* (Insecta: Thysanoptera) at different developmental stages: molecular evidence of *Wolbachia* endosymbiosis

Agnieszka Kaczmarczyk, Halina Kucharczyk, Marek Kucharczyk, Przemysław Kapusta, Jerzy Sell, Sylwia Zielińska

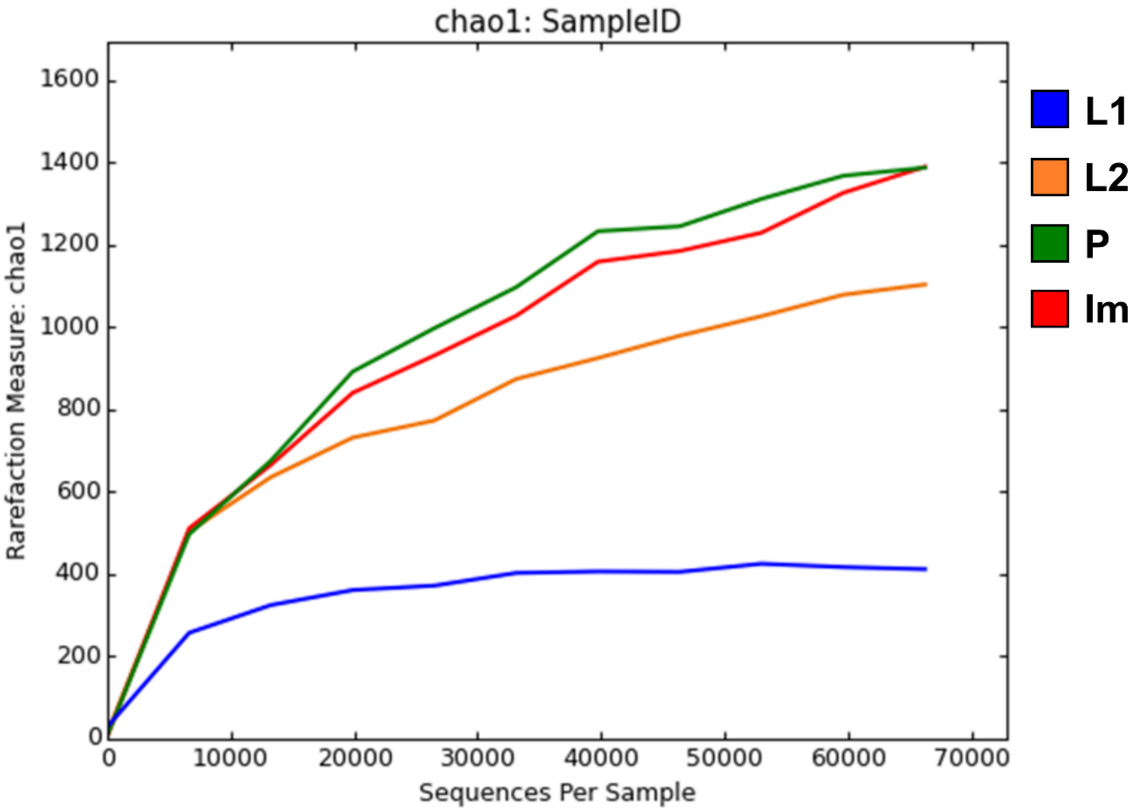

Supplementary Fig. S2. Plot showing alpha diversity (Chao1 richness estimator) variation across bacterial communities associated with four developmental stages of *H. carpathicus*. Abbreviations: L1 – first stage larvae, L2 – second stage larvae, P – pupae, Im – imago.
