# Supplementary material for: First insight into microbiome profile of fungivorous thrips Hoplothrips carpathicus (Insecta: Thysanoptera) at different developmental stages: molecular evidence of Wolbachia endosymbiosis
Source: Sci Rep. 2018 Sep 26;8:14376. doi: 10.1038/s41598-018-32747-x (PMC6158184; doi:10.1038/s41598-018-32747-x)
Supplement: Supplementary file 3 — Supplementary Table S1 [file 41598_2018_32747_MOESM3_ESM.pdf]

First insight into microbiome profile of fungivorous thrips *Hoplothrips carpathicus* (Insecta: Thysanoptera) at different developmental stages: molecular evidence of *Wolbachia* endosymbiosis

Agnieszka Kaczmarczyk, Halina Kucharczyk, Marek Kucharczyk, Przemysław Kapusta, Jerzy Sell, Sylwia Zielińska

Supplementary Table S1. Diversity indices calculated for microbial communities associated with four developmental stages of *H. carpathicus*.

ID	Shannon Index	Simpson Index
L1	6.36	0.98
L2	4.43	0.78
P	3.27	0.55
Im	3.44	0.69
Total	4.38	0.75

The ID abbreviations: L1 – first stage larvae, L2 – second stage larvae, P – pupae, Im – imago. Diversity indices represent the randomly selected subsets for each sample normalized to 66,241 sequences.
