# Supplementary material for: First insight into microbiome profile of fungivorous thrips Hoplothrips carpathicus (Insecta: Thysanoptera) at different developmental stages: molecular evidence of Wolbachia endosymbiosis
Source: Sci Rep. 2018 Sep 26;8:14376. doi: 10.1038/s41598-018-32747-x (PMC6158184; doi:10.1038/s41598-018-32747-x)
Supplement: Supplementary file 5 — Supplementary Fig. S3 [file 41598_2018_32747_MOESM5_ESM.zip › Supplementary_Figure_S2/Supplementary-Figure-S2-Im-resubmission.html]

Javascript must be enabled to view this page.

magnitude

 1.00000000000031

 .000828550847658

 .000828550847658

 .000828550847658

 .000828550847658

 .000828550847658

 .000828550847658

 .000405820823343

 .000405820823343

 .000405820823343

 .000405820823343

 .000405820823343

 .000405820823343

 .998765628329309

 1.7619387413466E-03

 0

 .000771059564351

 .000771059564351

 0

 0

 3.787661017865E-04

 3.787661017865E-04

 .000304365617507

 7.44004842795E-05

 0

 .000591822034042

 .000591822034042

 .000114982566614

 2.02910411671E-05

 2.02910411671E-05

 0

 0

 0

 0

 0

 0

 0

 .172318285272023

 9.942610171892E-04

 9.942610171892E-04

 2.70547215562E-05

 .000111600726419

 0

 0

 .168476514811045

 .168476514811045

 .000443021065483

 .000443021065483

 0

 0

 .00274943607815

 .00274943607815

 0

 0

 0

 .00397028038837

 .00397028038837

 0

 0

 .00252285278511

 .00252285278511

 0

 0

 .000459930266455

 0

 6.08731235014E-05

 0

 6.08731235014E-05

 .00305380169565

 .00305380169565

 1.04160677990905E-03

 .00103484309952

 6.76368038905E-06

 0

 1.11499271213441E-02

 .00348667724055

 .000443021065483

 0

 .000114982566614

 .00450122929891

 .00109571622303

 4.73457627233E-05

 3.72002421398E-05

 .000480221307622

 .000186001210699

 .013720125669195

 .00342242227686

 0

 .00205277699808

 .000534330750735

 .00459592082436

 0

 .0056814915268

 .0056814915268

 0

 .002113650121577

 .000236728813617

 0

 .00187692130796

 1.8938305089347E-03

 .0013594997582

 3.04365617507E-05

 0

 0

 0

 .00350358644153

 .00350358644153

 7.676777241561E-04

 .00020967409206

 2.02910411671E-05

 .000537712590929

 6.76368038905E-06

 6.76368038905E-06

 .000916478692716

 4.88675908109452E-03

 .00194117627166

 .00294220096924

 .108966272908

 .108966272908

 5.07276029179E-05

 5.07276029179E-05

 0

 0

 0

 0

 0

 0

 .000923242373105

 .000923242373105

 .000923242373105

 .000923242373105

 .001924267070684

 .000517421549762

 .000517421549762

 .001406845520922

 .000923242373105

 .000923242373105

 .000158946489143

 3.72002421398E-05

 3.72002421398E-05

 3.72002421398E-05

 3.72002421398E-05

 3.72002421398E-05

 3.19110440755327E-02

 .000273929055756

 .000273929055756

 0

 0

 0

 0

 .000578294673263

 .000578294673263

 .000578294673263

 .000148800968559

 0

 .007825578210123

 .007825578210123

 0

 .000145419128365

 .000145419128365

 .007680159081758

 .00738255714464

 .000297601937118

 .019888602184006

 .019888602184006

 .019888602184006

 .0174841138057

 .00170444745804

 3.3446399523847E-03

 3.3446399523847E-03

 3.3446399523847E-03

 0

 1.69092009726E-05

 9.13096852521E-05

 0

 2.6378353517252E-04

 2.6378353517252E-04

 2.6378353517252E-04

 2.6378353517252E-04

 3.38184019452E-06

 .000260401694978

 0

 0

 1.0348430995247E-03

 1.0246975789411E-03

 0

 0

 .000662840678127

 .000662840678127

 0

 0

 .000662840678127

 0

 0

 0

 1.35273607781E-05

 0

 1.35273607781E-05

 0

 0

 0

 0

 0

 0

 0

 0

 0

 1.01455205836E-05

 1.01455205836E-05

 .001017933898551

 0

 0

 0

 0

 .001017933898551

 .000886042130965

 .000886042130965

 0

 .000131891767586

 .000131891767586

 .000131891767586

 6.2800772412299E-03

 4.7447217929166E-03

 1.3020084748916E-03

 0

 0

 .00053094891054

 .00053094891054

 1.01455205836E-05

 1.01455205836E-05

 0

 0

 0

 0

 .000760914043768

 0

 0

 .000760914043768

 0

 0

 .003442713318025

 0

 0

 .000101455205836

 0

 0

 0

 .000771059564351

 0

 .000355093220425

 .000355093220425

 .00134597239742

 0

 .00134597239742

 1.5353554483133E-03

 1.5353554483133E-03

 6.76368038905E-05

 3.04365617507E-05

 0

 2.976019371176E-04

 1.01455205836E-05

 .000287456416534

 3.38184019452E-05

 .000537712590929

 0

 .000537712590929

 .000598585714431

 0

 .000598585714431

 0

 0

 0

 0

 0

 0

 0

 0

 0

 0

 0

 0

 0

 0

 0

 0

 0

 0

 0

 0

 0

 0

 0

 0

 0

 .000480221307622

 .000226583293033

 0

 .000226583293033

 .000706804600655

 .000706804600655

 .000706804600655

 2.1711414048814E-03

 0

 0

 2.1542322039088E-03

 .00211026828138

 0

 0

 .00211026828138

 4.39639225288E-05

 4.39639225288E-05

 4.39639225288E-05

 0

 0

 0

 1.69092009726E-05

 1.69092009726E-05

 1.69092009726E-05

 1.69092009726E-05

 0

 .779852348857137

 .59427725002315

 .006344332204926

 .006344332204926

 0

 0

 .000273929055756

 .00310114745838

 0

 .01943205375774

 .00103822493972

 .00103822493972

 0

 0

 1.9648491530176E-03

 .000439639225288

 0

 .00116673486711

 1.69092009726E-05

 .003699733172813

 .000158946489143

 .00354078668367

 1.6976837776517E-03

 .00167401089629

 0

 2.36728813617E-05

 .00103484309952

 .00103484309952

 3.111292978967E-04

 9.46915254467E-05

 .000165710169532

 .000165710169532

 0

 .003794424698259

 .000368620581203

 .000213055932255

 .000771059564351

 0

 0

 .00126819007295

 .00126819007295

 0

 1.02807941913812E-03

 1.02807941913812E-03

 .00101455205836

 3.38184019452E-06

 0

 1.01455205836E-05

 1.53873728850822E-03

 1.09909806322022E-03

 0

 0

 0

 3.38184019452E-06

 5.74912833069E-05

 4.39639225288E-05

 .000439639225288

 .561138597957

 .561138597957

 .561138597957

 4.7616309938922E-03

 0

 4.7616309938922E-03

 0

 .000429493704704

 .000111600726419

 .000923242373105

 .00304027433488

 .000229965133228

 3.82317033990639E-02

 6.76368038905E-06

 6.76368038905E-06

 6.76368038905E-06

 3.81133389922556E-02

 9.029513319378E-04

 .000419348184121

 3.04365617507E-05

 8.45460048631E-05

 .000558003632096

 .000429493704704

 0

 3.18975167147268E-02

 .0013594997582

 3.38184019452E-05

 3.38184019452E-06

 .0182619370504

 0

 3.38184019452E-06

 0

 0

 1.69092009726E-05

 0

 .00255328934687

 .004754867313495

 0

 0

 .00390602542467

 .000490366828206

 0

 0

 0

 0

 9.13096852521E-05

 0

 0

 0

 0

 0

 0

 2.02910411671E-05

 9.536789348554E-04

 3.38184019452E-05

 3.38184019452E-05

 3.38184019452E-05

 9.80733656412E-05

 9.80733656412E-05

 9.80733656412E-05

 0

 .000453166586066

 2.70547215562E-05

 3.72002421398E-05

 .000368620581203

 0

 .000368620581203

 0

 0

 0

 0

 .146389716500068

 0

 0

 .000297601937118

 0

 0

 0

 .000297601937118

 .000297601937118

 0

 0

 4.31522808821605E-03

 4.31522808821605E-03

 .00218128692547

 0

 6.76368038905E-06

 0

 0

 .000199528571477

 3.720024213972E-05

 3.720024213972E-05

 3.38184019452E-05

 .000784586925129

 .000784586925129

 .000784586925129

 0

 0

 0

 0

 .136281396158628

 1.14610564192391E-02

 6.76368038905E-06

 .00141360920131

 .00668927990477

 .00335140363277

 .124820339739389

 0

 .124813576059

 0

 0

 4.6737031488367E-03

 .000503894188984

 4.1698089598527E-03

 .000186001210699

 .000236728813617

 .00014203728817

 2.36728813617E-05

 0

 .0029050007271

 3.686205812028E-04

 5.74912833069E-05

 3.111292978959E-04

 .000253638014589

 3.348021792574E-04

 3.348021792574E-04

 3.348021792574E-04

 3.348021792574E-04

 4.05820823343E-05

 8.45460048631E-05

 0

 0

 0

 0

 0

 0

 0

 0

 0

 0

 0

 0

 0

 0

 0
