# Supplementary material for: First insight into microbiome profile of fungivorous thrips Hoplothrips carpathicus (Insecta: Thysanoptera) at different developmental stages: molecular evidence of Wolbachia endosymbiosis
Source: Sci Rep. 2018 Sep 26;8:14376. doi: 10.1038/s41598-018-32747-x (PMC6158184; doi:10.1038/s41598-018-32747-x)
Supplement: Supplementary file 5 — Supplementary Fig. S3 [file 41598_2018_32747_MOESM5_ESM.zip › Supplementary_Figure_S2/Supplementary-Figure-S2-L1-resubmission.html]

Javascript must be enabled to view this page.

magnitude

 .999999999999985

 .00202291632071

 .00202291632071

 .00202291632071

 .00202291632071

 .00202291632071

 .00202291632071

 0

 0

 0

 0

 0

 0

 .997977083679275

 .02453163448624

 0

 .00285321779563

 .00285321779563

 0

 0

 0

 0

 0

 0

 0

 0

 0

 0

 .00312495282378

 .00312495282378

 .01855346386683

 .00558566446763

 .0129677993992

 0

 0

 0

 0

 .343367400854499

 .00176627768301

 .00176627768301

 0

 0

 0

 0

 .286499298017884

 .285638803762061

 .000256638637702

 .000256638637702

 0

 0

 0

 0

 .0206216693589

 0

 .0206216693589

 .0365936504582

 .0365936504582

 0

 0

 0

 0

 .00300418170016

 .00300418170016

 .000015096390453

 .000015096390453

 0

 0

 0

 0

 0

 .02927190108841

 .0205310910161

 0

 0

 .000785012303558

 0

 0

 0

 0

 0

 .000785012303558

 0

 0

 0

 0

 .018719524161773

 .00141906070259

 .00182666324482

 .00203801271116

 0

 .00297398891925

 .0104467021935

 .00170589212119

 .00170589212119

 .000603855618122

 .002807928624263

 .000015096390453

 0

 .00279283223381

 .00209839827297

 0

 0

 0

 0

 0

 .0729004694977

 .0729004694977

 0

 0

 0

 0

 0

 0

 .0281698645854

 .038118385894

 .0271433100346

 .0109750758594

 .0249392370284

 .0249392370284

 0

 0

 .000860494255823

 .000860494255823

 .000860494255823

 0

 0

 0

 .0432662550384

 .0432662550384

 .0432662550384

 .0432662550384

 .011835570115205

 .0110656542021

 .0110656542021

 .000769915913105

 .000769915913105

 .000769915913105

 0

 0

 0

 0

 0

 0

 1.9776271493441E-03

 .000015096390453

 .000015096390453

 0

 0

 .000015096390453

 0

 0

 0

 0

 0

 0

 .000618952008575

 .000618952008575

 0

 0

 0

 .000618952008575

 .000015096390453

 0

 1.3435787503161E-03

 1.3435787503161E-03

 3.01927809061E-05

 3.01927809061E-05

 0

 0

 0

 0

 0

 0

 0

 0

 0

 0

 0

 0

 0

 0

 0

 0

 .09969656255193

 .03127972101873

 .00795579776875

 .00795579776875

 .00187195241618

 .00187195241618

 0

 0

 .00187195241618

 0

 0

 .0104467021935

 0

 0

 0

 0

 .0183723071814

 .0183723071814

 .0183723071814

 .0183723071814

 .0032155311665

 .0223728506514

 .0039854470796

 .0039854470796

 .0204707054543

 .0204707054543

 3.3664950710261E-03

 0

 0

 0

 0

 3.3664950710261E-03

 3.3664950710261E-03

 3.3664950710261E-03

 3.01927809061E-05

 0

 0

 0

 .151341314291718

 .118823689255902

 4.79008469075322E-02

 .00185685602572

 .00185685602572

 0

 0

 0

 0

 0

 0

 6.03855618122E-05

 0

 .04598360532

 0

 0

 .04598360532

 0

 0

 .07092284234837

 0

 0

 0

 0

 0

 0

 .01431137814947

 .0122431726574

 .0122431726574

 .0122431726574

 .0443682915415

 0

 .0443682915415

 3.25176250358161E-02

 3.25176250358161E-02

 0

 0

 0

 .00211349466343

 .00211349466343

 0

 0

 .02910584079342

 .00158512099757

 .00175118129255

 1.2982895789661E-03

 0

 0

 .00126809679806

 3.01927809061E-05

 0

 0

 0

 0

 0

 0

 .012696064371

 0

 .012696064371

 0

 0

 0

 .012696064371

 0

 0

 0

 0

 0

 0

 0

 0

 0

 0

 0

 0

 0

 0

 0

 0

 0

 .01532283630988

 0

 0

 .01532283630988

 .00264186832928

 0

 0

 .00264186832928

 .0126809679806

 .0126809679806

 .0126809679806

 0

 0

 0

 0

 0

 0

 0

 0

 .327108588336397

 .146148155975803

 .000966168988995

 .000966168988995

 0

 0

 0

 0

 0

 9.56205371295404E-02

 0

 0

 0

 0

 2.69923461300183E-02

 6.03855618122E-05

 0

 0

 3.01927809061E-05

 0

 0

 0

 .000015096390453

 0

 .000015096390453

 0

 4.11225675941061E-02

 .0410923748132

 0

 0

 0

 0

 0

 .024924140637953

 .0249090442475

 0

 .000015096390453

 .0012530004076

 .0012530004076

 0

 0

 0

 .013783004483606

 .013783004483606

 .000015096390453

 .0137528117027

 0

 .000015096390453

 .02216150118504

 .02216150118504

 .00689905043704

 0

 0

 0

 0

 0

 0

 .012530004076

 .012530004076

 .012530004076

 1.0869401126221E-03

 0

 1.0718437221691E-03

 0

 .000015096390453

 0

 0

 .00102655455081

 0

 7.68859165773322E-02

 0

 0

 0

 6.77073111818822E-02

 0

 0

 0

 0

 2.08632116061122E-02

 6.03855618122E-05

 0

 .03295542035896

 .00252109720566

 .0106429552694

 0

 0

 0

 0

 0

 0

 0

 .0037288084419

 0

 .01388867921681

 0

 0

 0

 .00276263945291

 0

 0

 0

 0

 0

 0

 0

 0

 0

 0

 0

 .00917860539545

 .00501200163041

 0

 0

 0

 0

 0

 0

 0

 .00501200163041

 0

 0

 0

 0

 0

 0

 0

 0

 0

 .099062514152851

 .00575172476261

 .00575172476261

 .00357784453737

 0

 0

 0

 .00357784453737

 .00357784453737

 0

 0

 .027037635301381

 .027037635301381

 .000271735028155

 .00267206111019

 .000015096390453

 .0206669585302

 0

 .000015096390453

 0

 0

 0

 .00640086955209

 .00640086955209

 .00640086955209

 0

 0

 0

 0

 .044489062665083

 .007125496293833

 0

 .000905783427183

 0

 .00621971286665

 .03736356637125

 .00966168988995

 .0277018764813

 .000800108694011

 .000800108694011

 .011005268640306

 .0109750758594

 .000030192780906

 0

 0

 0

 0

 0

 .000015096390453

 0

 0

 0

 0

 .01856856025724

 0

 0

 0

 0

 0

 .01856856025724

 .01856856025724

 .00889177397684

 0

 0

 0

 0

 0

 0

 0

 0

 0

 0

 0

 0
