# Supplementary material for: First insight into microbiome profile of fungivorous thrips Hoplothrips carpathicus (Insecta: Thysanoptera) at different developmental stages: molecular evidence of Wolbachia endosymbiosis
Source: Sci Rep. 2018 Sep 26;8:14376. doi: 10.1038/s41598-018-32747-x (PMC6158184; doi:10.1038/s41598-018-32747-x)
Supplement: Supplementary file 5 — Supplementary Fig. S3 [file 41598_2018_32747_MOESM5_ESM.zip › Supplementary_Figure_S2/Supplementary-Figure-S2-L2-resubmission.html]

Javascript must be enabled to view this page.

magnitude

 .999999999999699

 .00197385451721

 .00197385451721

 .00197385451721

 .00197385451721

 .00197385451721

 .00197385451721

 .000212126441741

 .000212126441741

 .000212126441741

 .000212126441741

 .000212126441741

 .000212126441741

 .997814019040748

 1.8803750683133E-03

 7.19072683867E-06

 .000413466793223

 .000413466793223

 0

 .000413466793223

 0

 0

 0

 0

 4.67397244513E-05

 .000262461529611

 .000262461529611

 0

 3.59536341933E-06

 3.59536341933E-06

 .00114692093077

 0

 0

 .00114692093077

 .00114692093077

 0

 0

 .56939770471998

 1.9810452440513E-03

 1.9810452440513E-03

 0

 0

 4.67397244513E-05

 4.67397244513E-05

 .565281013604843

 .564871142175039

 .000675928322835

 .000675928322835

 0

 0

 .000877268674318

 .000877268674318

 2.121264417404E-04

 5.75258147094E-05

 .000154600627031

 .00527799349958

 .00527799349958

 .000115051629419

 .000115051629419

 .00162869962896

 .00162869962896

 0

 0

 5.752581470934E-04

 .00048537406161

 .000330773434579

 .000319987344321

 0

 0

 0

 .00133747519199

 .00101389248425

 .000201340351483

 1.43814536773E-05

 1.99578623407424E-02

 .00384344349527

 0

 .000136623809935

 0

 .0107645180775

 .00428207783243

 6.83119049674E-05

 .000165386717289

 0

 .00005393045129

 4.46544136681768E-02

 8.98840854834E-05

 .000553685966578

 .00151724336296

 0

 .0357882474761

 7.90979952254E-05

 .00763655190267

 .00763655190267

 .0006507607789

 5.27439813616867E-03

 .00147769436535

 7.19072683867E-06

 .00378951304398

 2.70011792791733E-03

 3.59536341933E-06

 .000204935714902

 .000021572180516

 0

 0

 .0060258290908

 .0060258290908

 .002078120056374

 .000765812408318

 .000021572180516

 .00129073546754

 0

 0

 .00289067218914

 .002800788103668

 .0016358903558

 .00115411165761

 .452595133316

 .452595133316

 0

 0

 .000409871429804

 .000409871429804

 .000409871429804

 .000140219173354

 .000140219173354

 .000140219173354

 .000589639600771

 .000589639600771

 .000589639600771

 .000589639600771

 1.4057870969601E-03

 2.87629073547E-05

 2.87629073547E-05

 1.3770241896054E-03

 .001075013662381

 .000614807144706

 5.75258147094E-05

 0

 0

 0

 0

 0

 1.44857192165004E-02

 .001182874564961

 .001182874564961

 .000032358270774

 .000032358270774

 .000934794489027

 .000248080075934

 .002074524692954

 .002074524692954

 .002074524692954

 .000575258147094

 .00139140564328

 3.9980441223034E-03

 3.9980441223034E-03

 7.19072683867E-05

 0

 0

 3.9261368539167E-03

 .00385422958553

 7.19072683867E-05

 .006237955532545

 .006237955532545

 .005820893375902

 .000744240227802

 .00406635602727

 .000992320303737

 .000992320303737

 .000370322432192

 .000179768170967

 0

 0

 .000621997871545

 0

 0

 0

 0

 0

 0

 .00075502631806

 .00075502631806

 .004641614174365

 .002516754393533

 .00107501366238

 .00107501366238

 .000212126441741

 .000212126441741

 0

 0

 0

 0

 .000212126441741

 0

 .00080895676935

 .00005393045129

 .00075502631806

 .000639974688642

 0

 0

 0

 0

 0

 0

 0

 0

 .00148488509219

 .00148488509219

 1.76028993010973E-02

 1.43814536773E-05

 1.43814536773E-05

 1.43814536773E-05

 1.43814536773E-05

 .01758851784742

 .01758851784742

 .01758851784742

 .0124902925188

 0

 0

 0

 .013151839387932

 .013151839387932

 5.20249086778334E-03

 7.19072683867E-06

 7.19072683867E-06

 1.53162481663867E-03

 .0015244340898

 .000553685966578

 .000553685966578

 .000532113786061

 .000532113786061

 0

 0

 .002567089481409

 0

 .000111456265999

 .00245563321541

 0

 0

 7.9493485201487E-03

 8.26933586447E-05

 8.26933586447E-05

 0

 0

 .000043144361032

 .000043144361032

 0

 0

 0

 0

 .007823510800472

 .000258866166192

 .00756464463428

 0

 0

 0

 0

 0

 0

 0

 0

 0

 0

 0

 0

 0

 0

 0

 0

 0

 7.19072683867E-06

 7.19072683867E-06

 7.19072683867E-06

 7.19072683867E-06

 0

 7.19072683867E-06

 6.112117812867E-05

 .000032358270774

 2.876290735467E-05

 0

 0

 0

 0

 7.19072683867E-06

 .000586044237351

 .000586044237351

 .000586044237351

 .00005393045129

 .000532113786061

 0

 0

 0

 0

 0

 .000575258147094

 .000575258147094

 0

 .00105344148186

 .00105344148186

 .00105344148186

 4.817786981907E-04

 .00010786090258

 .00010786090258

 2.624615296117E-04

 .000190554261225

 0

 0

 .000190554261225

 7.19072683867E-05

 7.19072683867E-05

 7.19072683867E-05

 0

 0

 0

 .000111456265999

 .000111456265999

 .000111456265999

 .000111456265999

 0

 .370502200362088

 .177269393389883

 3.4551442459847E-03

 3.4551442459847E-03

 .000118646992838

 1.79768170967E-05

 0

 0

 0

 1.69269709782434E-02

 3.59536341933E-06

 3.59536341933E-06

 0

 0

 2.3837259470237E-03

 .000197744988063

 6.11211781287E-05

 .00132309373832

 0

 .00153522018006

 0

 .00153522018006

 .00260663847902

 .0020637386027

 .000179768170967

 .000363131705353

 .00562674375126

 .00562674375126

 7.909799522537E-04

 .000708286593609

 2.6605689303067E-04

 .000258866166192

 7.19072683867E-06

 0

 0

 0

 0

 0

 0

 .0029374119136

 .0029374119136

 0

 3.66151810625353E-02

 3.66151810625353E-02

 .00339402306785

 .0296905111169

 .00111096729657

 .00239451203728

 7.48195127563233E-03

 6.48244024505733E-03

 3.59536341933E-06

 0

 .000165386717289

 .00178330025599

 0

 .000165386717289

 .000999511030575

 .106749935283

 .106749935283

 .106749935283

 6.0402105444877E-03

 .000258866166192

 4.4906089107557E-03

 .000010786090258

 .00158915063135

 .000506946242126

 8.26933586447E-05

 .000938389852446

 .000100670175741

 .053614059309135

 0

 0

 0

 5.29165588057843E-02

 .00306324963327

 .00306324963327

 0

 0

 .006680185233127

 .006284695257

 0

 4.12100555124293E-02

 .00187318434147

 .000302010527224

 .000291224436966

 .00081255213277

 3.95489976127E-05

 1.43814536773E-05

 7.19072683867E-06

 7.19072683867E-06

 0

 0

 .000582448873932

 .001963068426958

 .000190554261225

 .000129433083096

 0

 .000176172807547

 .000251675439353

 .000251675439353

 .000251675439353

 .000287629073547

 0

 0

 0

 8.26933586447E-05

 8.26933586447E-05

 0

 8.26933586447E-05

 .000075502631806

 9.743434866393E-04

 .000179768170967

 .000179768170967

 .000179768170967

 0

 0

 0

 3.59536341933E-05

 .00037751315903

 0

 0

 .000381108522449

 .000381108522449

 0

 .000075502631806

 .000075502631806

 .000075502631806

 .000075502631806

 .138568901544624

 1.79768170967E-05

 1.79768170967E-05

 4.278482469004E-04

 0

 7.90979952254E-05

 7.90979952254E-05

 .000240889349095

 .000240889349095

 .00010786090258

 .00010786090258

 8.33225472431366E-02

 8.33225472431366E-02

 .0324984899474

 0

 .00733454137544

 3.59536341933E-06

 3.59536341933E-06

 .000661546869158

 6.11211781287E-05

 0

 0

 .00220755313947

 .00220755313947

 .00220755313947

 .000151005263612

 0

 0

 0

 .030970460494124

 .005601576207324

 0

 .00298415163805

 .000816147496189

 .00161431817528

 .0253688842868

 0

 .0253688842868

 0

 0

 .021410389162156

 .000614807144706

 .02079558201745

 .00026965225645

 .0030488681796

 0

 .00225788822734

 0

 .0122889521673

 1.797681709663E-04

 4.67397244513E-05

 .000133028446515

 0

 9.024362182527E-04

 9.34794489027E-05

 9.34794489027E-05

 9.34794489027E-05

 0

 0

 .000506946242126

 .000506946242126

 0

 .000302010527224

 .000302010527224

 .000302010527224

 0

 .000302010527224

 .00154960163373

 .00154960163373

 .00154960163373

 0

 0

 .00154960163373

 .00154960163373
