# Supplementary material for: First insight into microbiome profile of fungivorous thrips Hoplothrips carpathicus (Insecta: Thysanoptera) at different developmental stages: molecular evidence of Wolbachia endosymbiosis
Source: Sci Rep. 2018 Sep 26;8:14376. doi: 10.1038/s41598-018-32747-x (PMC6158184; doi:10.1038/s41598-018-32747-x)
Supplement: Supplementary file 5 — Supplementary Fig. S3 [file 41598_2018_32747_MOESM5_ESM.zip › Supplementary_Figure_S2/Supplementary-Figure-S2-P-resubmission.html]

Javascript must be enabled to view this page.

magnitude

 1.00000000000037

 1.81662942576E-05

 1.81662942576E-05

 1.81662942576E-05

 1.81662942576E-05

 1.81662942576E-05

 1.81662942576E-05

 0

 0

 0

 0

 0

 0

 .999981833706116

 .007420931204242

 0

 .000758442785256

 .000758442785256

 .000458698930005

 0

 .00655349065344

 .00655349065344

 .00655349065344

 0

 0

 0

 0

 0

 0

 0

 0

 0

 0

 0

 0

 .000108997765546

 .000108997765546

 7.76563663778722E-02

 .006285537813146

 .006285537813146

 .000840191109416

 0

 0

 0

 6.79464820971552E-02

 6.79464820971552E-02

 .000440532635748

 .000440532635748

 .00109451922902

 .00109451922902

 .000376950605846

 .000376950605846

 .000563155121987

 0

 .000563155121987

 .0100595854452

 .0100595854452

 0

 0

 0

 0

 0

 0

 0

 0

 0

 0

 0

 .000794775373772

 .000794775373772

 .002061874398239

 .00171217323378

 0

 0

 .005009355641541

 .000472323650698

 .000417824767926

 0

 0

 .00102185405199

 .000976438316348

 .000853815830109

 0

 0

 0

 7.06214689265641E-03

 4.54157356441E-06

 0

 .00477319381619

 0

 .00122168328883

 .00104002034625

 .00317910149509

 .00317910149509

 .00134430577506

 8.4927425654482E-04

 9.08314712882E-06

 0

 .000840191109416

 .000921939433575

 .00011353933911

 0

 0

 .000022707867822

 .000022707867822

 .021336312605592

 .0211183170745

 .006894108670775

 .000694860755355

 0

 .00619924791542

 0

 0

 .000272494413865

 .000867440550802

 .000490489944956

 .000376950605846

 .00367413301361

 .00367413301361

 0

 0

 0

 0

 0

 .00105818664051

 .00105818664051

 .00105818664051

 .00118080912675

 .00118080912675

 .00118080912675

 .00118080912675

 .001185350700311

 0

 0

 .001185350700311

 .000590404563373

 0

 0

 0

 0

 0

 0

 0

 .045838101985617

 .000326993296637

 .000326993296637

 0

 0

 .000326993296637

 0

 .000726651770305

 .000726651770305

 .000726651770305

 .000726651770305

 0

 .002279869929334

 .002279869929334

 0

 .00038149217941

 .00038149217941

 .001898377749924

 .00158500917398

 0

 .038408087634241

 .038408087634241

 .038408087634241

 .0275173942268

 .010436536051

 .0040964993551

 .0040964993551

 .0040964993551

 0

 0

 0

 0

 4.54157356441E-06

 4.54157356441E-06

 4.54157356441E-06

 4.54157356441E-06

 4.54157356441E-06

 0

 .00155775973259

 .00155775973259

 .004532490417276

 .004051083619449

 .000172579795448

 .000172579795448

 .003419804893996

 .003419804893996

 .00104456191981

 .000971896742783

 .000962813595655

 .000440532635748

 0

 0

 0

 0

 0

 0

 .000299743855251

 .000299743855251

 .000299743855251

 .000299743855251

 0

 0

 0

 0

 .000181662942576

 .000181662942576

 .000676694461097

 0

 0

 0

 0

 .000676694461097

 .000435991062183

 .000435991062183

 0

 .000240703398914

 .000240703398914

 .000240703398914

 3.31353207259374E-02

 2.33527712681954E-02

 1.15537631478564E-02

 .000217995531092

 .000217995531092

 .00377858920559

 .00377858920559

 0

 0

 .000208912383963

 .000208912383963

 2.7703598742941E-04

 .000272494413865

 .007071230039782

 .000217995531092

 0

 .00685323450869

 .00161225861537

 .00161225861537

 .010186749504969

 0

 0

 .00104910349338

 .00104910349338

 0

 0

 0

 0

 0

 0

 .009137646011589

 .000390575326539

 .00874707068505

 .009782549457742

 .009782549457742

 0

 0

 .00793867059059

 .00101731247843

 .00101731247843

 0

 0

 0

 0

 0

 .000826566388722

 .000590404563373

 .000236161825349

 0

 0

 .00107635293476

 .00107635293476

 .00107635293476

 .00107635293476

 .00107635293476

 0

 .000172579795448

 0

 .000172579795448

 .000172579795448

 .000172579795448

 .000172579795448

 0

 0

 .000445074209312

 .000445074209312

 .000445074209312

 0

 0

 .000131705633368

 .000131705633368

 .000313368575944

 .000313368575944

 0

 0

 0

 0

 0

 0

 0

 1.87112830853682E-03

 0

 0

 1.41697095209582E-03

 4.7686522426282E-04

 9.08314712882E-06

 9.08314712882E-06

 .000467782077134

 .000585862989809

 .000585862989809

 0

 .000354242738024

 .000354242738024

 .000354242738024

 .000454157356441

 .000454157356441

 .000454157356441

 .000331534870202

 .000122622486239

 .822029356731804

 .731552128181666

 .000317910149509

 .000317910149509

 0

 0

 0

 0

 .000585862989809

 9.56455392663844E-03

 1.36247206932E-05

 1.36247206932E-05

 .00042236634149

 .00042236634149

 1.78483841080982E-03

 4.54157356441E-06

 0

 4.54157356441E-06

 .000299743855251

 .00124439115665

 0

 .00124439115665

 .00117626755318

 .00117626755318

 0

 0

 .00116718440605

 .00116718440605

 .000104456191981

 0

 9.1285628644641E-04

 4.54157356441E-06

 0

 8.1294166802901E-04

 .000790233800207

 0

 1.81662942576E-05

 0

 0

 .001035478772685

 .00069031918179

 .000345159590895

 .003133685759445

 .003133685759445

 0

 .00095827202209

 .00163496648319

 0

 .014028920740447

 .014028920740447

 0

 .000099914618417

 .00165313277744

 .00545442985085

 0

 0

 0

 .699456827802

 .699456827802

 .699456827802

 4.4643668138175E-03

 .000277035987429

 4.1873308263885E-03

 .000022707867822

 .00064944501971

 .00030882700238

 0

 .00244790815122

 .00073119334387

 2.14816429596795E-02

 0

 0

 0

 2.00646720075845E-02

 .00273402728577

 .00273402728577

 0

 0

 .001230766435955

 .000390575326539

 .000372409032282

 1.33885588679075E-02

 .00167584064527

 4.54157356441E-05

 0

 4.54157356441E-06

 0

 0

 .000254328119607

 0

 0

 0

 .000022707867822

 .002711319417952

 0

 0

 .000331534870202

 .00136701364289

 0

 0

 0

 0

 .00042236634149

 .000345159590895

 .000345159590895

 .00064944501971

 .00064944501971

 .00064944501971

 0

 0

 .001439678819917

 0

 0

 0

 .000567696695551

 .000567696695551

 0

 0

 0

 0

 0

 .000871982124366

 0

 .000871982124366

 0

 0

 0

 0

 6.75559067705414E-02

 4.54157356441E-06

 4.54157356441E-06

 .000199829236834

 .000199829236834

 0

 0

 0

 0

 0

 0

 3.24949588533312E-02

 3.24949588533312E-02

 .00148963612913

 .000345159590895

 .000349701164459

 0

 1.36247206932E-05

 .000426907915054

 4.54157356441E-06

 4.54157356441E-06

 0

 .00182117099933

 .00182117099933

 .00182117099933

 .000199829236834

 .000304285428815

 .000304285428815

 .000304285428815

 2.73039402691944E-02

 2.77035987429441E-03

 0

 .00163496648319

 4.54157356441E-06

 .00113085181754

 .0245335803949

 0

 .0245335803949

 .000413283194361

 .000413283194361

 .004809526404713

 .00317001834796

 .001639508056753

 0

 .000254328119607

 .000108997765546

 .000594946136938

 .000395116900104

 0

 .001557759732589

 .00120805856813

 .000349701164459

 .000349701164459

 .000454157356441

 0

 0

 0

 0

 0

 0

 0

 0

 .000454157356441

 .000454157356441

 .000454157356441

 .000454157356441

 0

 .001553218159029

 .001553218159029

 .001553218159029

 .00104910349338

 .00104910349338

 .000504114665649

 .000504114665649
